# The role of intestinal microbiota in the pathogenesis of childhood asthma

**DOI:** 10.3389/fimmu.2026.1807925

**Published:** 2026-05-08

**Authors:** Lulu Zhang, Li Wu, Chunlin Shi, Qin Huang, Lu Zhan, Yan Zeng, Quanmin Deng

**Affiliations:** Department of Paediatrics, People’s Hospital of Deyang City, Deyang, Sichuan, China

**Keywords:** childhood asthma, gut microbiota, gut–lung axis, immune homeostasis, inflammation, microbial metabolites, mucosal immunity, Th17/Treg balance

## Abstract

Childhood asthma represents a multifactorial inflammatory disorder shaped by genetic predisposition, environmental exposures, and immune dysregulation. Growing evidence underscores the gut microbiota as a critical mediator linking early-life microbial colonization with long-term respiratory immune outcomes. Gut commensals influence key immunological processes—including Th1/Th2/Th17/Treg balance, dendritic cell maturation, and epithelial barrier integrity—thereby shaping host susceptibility to asthma. Moreover, microbial metabolites such as SCFAs, LPS, tryptophan derivatives, and secondary bile acids serve as potent immunoregulatory agents, capable of either promoting or attenuating airway inflammation. The gut–lung axis provides a conceptual and mechanistic framework through which intestinal microbial alterations influence pulmonary immunity. This review outlines how microbial dysbiosis disrupts immune homeostasis by affecting T cell subset differentiation, dendritic and epithelial cell function, mucosal immunity, and inflammatory signaling, offering novel insights into asthma pathogenesis and highlighting promising targets for microbiota-based prevention and therapeutic strategies.

## 1 Introduction

Asthma affects approximately 260 million people worldwide, especially in south Asia (1). Asthma is a respiratory disease driven by the interaction of genetic and environmental factors, involving inflammatory cells, airway structural cells, and cellular components (2–4). The pathogenesis of asthma is closely related to immune, neural, endocrine, and genetic factors. Emerging evidence highlights substantial differences in gut microbiota composition between individuals with asthma and healthy controls, suggesting a potential etiological link. The gut microbiota may influence asthma onset and progression through modulation of T lymphocyte subsets, dendritic cell activation, airway epithelial barrier integrity, mucosal immune responses, and associated signaling pathways (5, 6). Furthermore, microbial metabolites, such as short-chain fatty acids, lipopolysaccharides, tryptophan derivatives, and secondary bile acids, exert bidirectional immunomodulatory effects, acting either protectively or promotively in the context of asthma pathophysiology (7, 8).

The human gut microbiota comprises an estimated 100 trillion microorganisms, forming a complex and dynamic ecosystem essential for host health and immune regulation (9). Studies have demonstrated that the microbial diversity in the gut of children with asthma is markedly reduced during early postnatal periods (10, 11). Moreover, asthmatic children exhibit a notable depletion of beneficial commensals, such as *Bifidobacterium* and *Lactobacillus*, accompanied by an overrepresentation of potentially pathogenic taxa including *Escherichia coli*, *Streptococcus*, and *Staphylococcus* (12, 13). In a longitudinal analysis, Stokholm et al. (14) identified the association between increased childhood asthma risk and elevated levels of *Veillonella*. These findings underscore the crucial role of gut microbiota homeostasis in maintaining normal immune function (15). In light of this, this review summarizes the role and related mechanisms of gut microbiota and its metabolites in the pathogenesis of asthma.

## 2 Immune function of gut microbiota in childhood asthma pathogenesis

### 2.1 Modulation of T lymphocyte subsets

T lymphocytes, including T helper (Th) cells, regulatory T (Treg) cells, and cytotoxic T lymphocytes, are pivotal orchestrators of immune homeostasis. They are critically involved in immune surveillance, tolerance maintenance, and the suppression of autoimmune and neoplastic processes (16, 17). Multiple T cell subtypes contribute to the immunopathology of asthma, reflecting the complex interplay between adaptive immunity and airway inflammation (18). Th1 cells, by secreting cytokines such as TNF-β, IFN-γ, and IL-2, mediate macrophage-driven antimicrobial and antiviral immune responses (19).

Gut microbial signals critically influence the transcriptional circuitry that governs Th1/Th2 lineage commitment (20). Microbe-associated molecular patterns, including lipopolysaccharide, peptidoglycan, and flagellin, activate Toll-like receptors and related pattern-recognition receptors on dendritic cells, resulting in MyD88- and NF-κB-dependent production of IL-12 and type I interferons (21). IL-12 subsequently activates STAT4 signaling in naïve CD4^+^ T cells and induces expression of T-bet, the master transcription factor of the Th1 lineage, thereby enhancing IFN-γ secretion and suppressing excessive Th2 polarization (22). In contrast, epithelial injury caused by allergens or dysbiotic microbial metabolites promotes release of IL-25, IL-33, and thymic stromal lymphopoietin, which condition dendritic cells to favor IL-4-dominant responses (23, 24). IL-4 activates JAK1/JAK3–STAT6 signaling and upregulates GATA3, the central transcriptional regulator of Th2 differentiation, leading to enhanced production of IL-4, IL-5, and IL-13 (25, 26). These cytokines drive eosinophil recruitment, goblet-cell metaplasia, mucus hypersecretion, airway smooth muscle hyperreactivity, and IgE-mediated allergic inflammation (27). Thus, disruption of early-life microbial signaling may impair Th1 maturation while sustaining aberrant STAT6/GATA3-dependent Th2 immunity, thereby increasing susceptibility to childhood asthma (28, 29).

**Figure 1:**
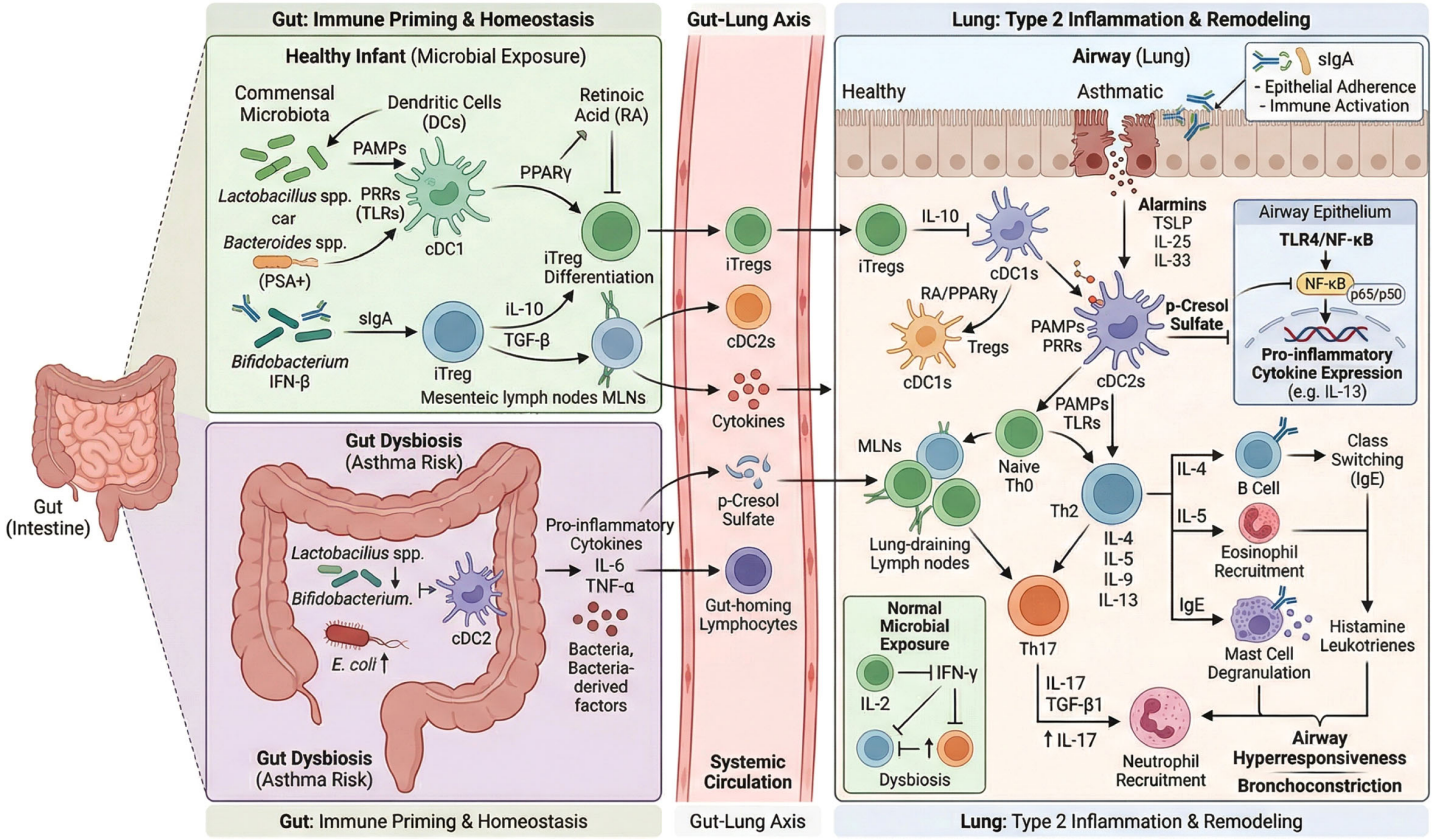
Immune function of gut microbiota in childhood asthma pathogenesis.

During fetal development, the immature immune system is skewed toward a Th2-dominant profile, presumably to reduce immunological conflict between the fetus and the maternal immune system. Postnatally, microbial exposure induces a shift toward Th1-mediated immunity via cytokines such as IL-2 and IFN-γ, thereby rebalancing the Th1/Th2 axis (27, 30). Perturbations in this transition are implicated in allergic predisposition. The gut microbiota profoundly influences this immunological balance. For instance, *Lactobacillus* species have been shown to suppress pro-inflammatory cytokines (IL-6 and TNF-α) and reduce the production of antigen-specific IgE (31). Additionally, *Bacteroides* spp. secrete polysaccharide A, which enhances Treg differentiation and thereby promotes immune tolerance by modulating Th1/Th2 responses (32). Probiotic supplementation may further sustain this equilibrium by enhancing Treg induction and regulating the expression of key immunomodulatory factors (33) (**Figure 1**).

### 2.2 Modulation of Th17 responses by gut microbiota in childhood asthma

Gut microbiota plays a pivotal role in orchestrating the differentiation and activation of Th17 cells (34). Experimental evidence reveals that oral administration of *Lactobacillus* significantly attenuates airway inflammation in murine models of asthma by downregulating pulmonary infiltration of granulocytes and suppressing Th2- and Th17-associated inflammatory cytokines (35, 36). Similarly, in breastfed infants receiving *Bifidobacterium* supplementation, a marked silencing of Th2- and Th17-related cytokines in the intestinal milieu has been observed, concomitant with enhanced production of IFN-β, a response that may confer protection against both gut and airway inflammation (37). Clinical observations further underscore the immunoregulatory role of gut microbiota. Jiang et al. (38) compared fecal microbiota composition and peripheral immune profiles between children with acute asthma exacerbation and healthy controls. Asthmatic children exhibited significantly lower levels of beneficial microbes such as *Lactobacillus* and *Bifidobacterium*, while harboring increased *Escherichia coli* abundance. Peripheral blood analysis revealed elevated levels of TGF-β1, IL-17, Th17 cell frequency, and Th17/Treg ratio in asthmatic patients, whereas Treg cell levels were markedly reduced compared to controls. These findings implicate gut dysbiosis in the promotion of Th17-driven inflammation, thus contributing to asthma onset and progression. Importantly, Th17 and Treg cells should not be considered completely separate and stable lineages, as they display substantial plasticity in response to inflammatory signals (39, 40). Under homeostatic conditions, TGF-β promotes FOXP3^+^ Treg differentiation, whereas in the presence of IL-6, this balance shifts toward STAT3/RORγt-mediated Th17 polarization, with partial loss of Treg suppressive function (41, 42). Gut microbiota critically regulates this process. Dysbiosis may increase IL-6 and IL-1β production, thereby favoring Th17 responses, while beneficial microbial metabolites such as SCFAs and tryptophan derivatives promote Treg stability, IL-10 production, and immune tolerance (43, 44). Thus, microbiota-dependent control of the Th17/Treg axis represents an important mechanism in childhood asthma pathogenesis.

Tregs function as key negative regulators of immune activation, maintaining immunological tolerance and preventing excessive allergic responses (45, 46). Two major subsets of Tregs have been identified: natural Tregs (nTregs), which originate from thymic differentiation of hematopoietic progenitors, and inducible Tregs (iTregs), which arise in peripheral lymphoid tissues upon antigen exposure. Both subsets exert immunosuppressive functions primarily through the secretion of anti-inflammatory cytokines such as IL-10 and TGF-β, thereby attenuating hyperreactive immune responses (47). Dysfunction of Treg populations has been directly associated with the pathogenesis of asthma and other allergic diseases (45, 48). Zhang et al. (49) established a gut microbiota-depleted mouse model by perinatal exposure to broad-spectrum ampicillin. Offspring of the treated mice demonstrated reduced microbial diversity and a notable deficiency in Treg accumulation, leading to Treg dysfunction and exacerbated airway inflammation. Consistent with these preclinical observations, researchers have observed low peripheral Treg levels in children undergoing acute asthma attacks (38). Notably, Treg cell frequency exhibited a positive correlation with fecal levels of beneficial probiotics and a negative correlation with *E. coli* abundance.

### 2.3 Regulation of dendritic cells and airway epithelial cells

Dendritic cells (DCs) are the most potent professional APCs, endowed with the capacity to efficiently capture, process, and present antigens to naïve T cells. Upon maturation, DCs play a pivotal role in initiating adaptive immune responses by activating T lymphocytes (50, 51). In the lung, type 1 DCs (cDC1s) which upon exposure to allergens can promote the differentiation of Tregs through retinoic acid and PPARγ signaling (52). The type 2 DCs (cDC2s) primarily drive the polarization of Th2 and Th17 cells (53). Both subsets are integral to the initiation, regulation, and maintenance of immune responses at mucosal surfaces. In parallel, airway epithelial cells constitute the first physical and immunological barrier of the respiratory tract. These cells maintain airway homeostasis by producing cytokines, chemokines, and remodeling factors that shape the local immune environment (54, 55).

Upon stimulation by aeroallergens, respiratory viruses, or microbial products, airway epithelial cells rapidly activate NF-κB, MAPK (ERK1/2, p38, JNK), and JAK/STAT signaling cascades, leading to transcriptional induction of multiple pro-inflammatory mediators. These include thymic stromal lymphopoietin (TSLP), IL-25, and IL-33, which act as epithelial-derived alarmins that strongly promote type 2 immunity by activating group 2 innate lymphoid cells (ILC2s), mast cells, and basophils, ultimately enhancing IL-4, IL-5, and IL-13 production (56, 57). IL-13 further drives goblet cell metaplasia, mucus hypersecretion, and airway hyperresponsiveness (58, 59). Airway epithelial cells also secrete chemokines such as CCL20, CXCL8, CCL17, and CCL22, which recruit dendritic cells, eosinophils, neutrophils, and Th2 lymphocytes into inflamed airways, thereby amplifying chronic inflammation (60, 61). In addition, epithelial-derived GM-CSF enhances DC survival and antigen-presenting capacity, facilitating allergen sensitization (62, 63). Persistent epithelial injury induces TGF-β, epidermal growth factor receptor (EGFR), and YAP/TAZ signaling, promoting subepithelial fibrosis, smooth muscle remodeling, and irreversible airway structural changes (64, 65). Pattern recognition receptors (PRRs), such as TLRs and formyl peptide receptors—expressed on both epithelial cells and DCs, recognize PAMPs, which are conserved structural motifs on potentially pathogenic microbes (66).

Within the intestinal tract, DCs utilize PRRs to discriminate between pathogenic and commensal microbes via recognition of PAMPs. This process governs downstream immune responses or the induction of immune tolerance, particularly by directing Th0 cell differentiation into distinct effector lineages, including Th17, Th2, Th1, or Treg subsets (67, 68). Notably, proinflammatory cytokines secreted by pathogenic microbes in the gut can migrate to the pulmonary environment via gut-homing lymphocytes and systemic circulation, thus establishing a gut–lung immunological axis (69). When airway epithelial cells are damaged by environmental allergens or microbial insults, they release a repertoire of epithelial-derived alarmins and chemotactic mediators such as thymic stromal lymphopoietin, IL-25, and IL-33. These cytokines facilitate the differentiation of Th0 cells into Th2 cells, thereby enhancing type 2 inflammation and increasing susceptibility to asthma (70).

### 2.4 Regulation of mucosal immunity

The mucosal immune system constitutes the first immunological interface between the host and the external environment, where intestinal microorganisms continuously interact with epithelial and immune cells to shape immune tolerance and inflammatory responsiveness (71, 72). Among mucosal effector molecules, immunoglobulin A (IgA), particularly secretory IgA (sIgA), is the dominant antibody isotype and serves as a central mediator of host–microbiota mutualism (73, 74). sIgA limits microbial encroachment by coating luminal bacteria, blocking epithelial adhesion, neutralizing toxins and microbial antigens, and promoting immune exclusion without inducing excessive complement-mediated tissue injury (75, 76). Through these mechanisms, IgA preserves microbial diversity while restraining the expansion of pro-inflammatory pathobionts, thereby maintaining intestinal immune homeostasis. Impairment of IgA responses may contribute to allergic sensitization and asthma susceptibility. Reduced sIgA levels in the saliva and intestinal secretions of infants, as well as a decreased proportion of IgA-coated fecal bacteria, have been associated with an increased risk of childhood allergic disease and asthma (77–80). Insufficient IgA coating permits greater epithelial contact by microbial products and dietary antigens, enhancing barrier disruption and facilitating the release of inflammatory mediators. This may promote dendritic cell activation and skew naïve CD4^+^ T-cell differentiation toward Th2 and Th17 phenotypes, characterized by increased IL-4, IL-5, IL-13, IL-17A, and IL-17F, while weakening regulatory circuits mediated by IL-10 and Foxp3^+^ Treg cells (81). Consequently, defective IgA-mediated mucosal containment may create a permissive immunological background for eosinophilic inflammation, airway hyperresponsiveness, and persistent allergic immune activation in childhood asthma.

### 2.5 Modulation of inflammatory signaling pathways

As central regulators of inflammation, multiple signaling pathways are critically involved in the pathogenesis and progression of asthma. These pathways exert dual regulatory functions: they can trigger inflammatory cascades and exacerbate airway hyperresponsiveness, or conversely, suppress chemokine expression and mitigate airway remodeling (82, 83). Emerging evidence suggests that specific probiotic strains can attenuate pulmonary oxidative stress and inflammation by downregulating the TLR4/NF-κB signaling axis (84). This modulation dampens the expression of proinflammatory cytokines and reduces immune cell infiltration, thereby alleviating asthma symptoms. Moreover, certain gut microbiota enhances L-tyrosine metabolism, leading to increased production of its downstream metabolite, p-cresol sulfate. This metabolite inhibits the synthesis of carbon tetrachloride by airway epithelial cells and simultaneously promotes dissociation of the EGFR/TLR4 signaling complex. As a result, downstream recruitment and activation of eosinophils, neutrophils, dendritic cells, and chemokine release are attenuated, along with decreased levels of IL-13, a cytokine central to the pathophysiology of allergic airway inflammation (85).

## 3 Gut microbial metabolites in the pathogenesis of childhood asthma

### 3.1 Short-chain fatty acids

An expanding body of evidence implicates several gut microbiota–derived metabolites, including SCFAs, lipopolysaccharides, D-tryptophan, and secondary bile acids, in the immunopathogenesis of childhood asthma. Among these, SCFAs have garnered particular attention due to their multifaceted roles in immune regulation and epithelial homeostasis. SCFAs are generated by gut microbiota through the anaerobic fermentation of indigestible dietary fibers (86, 87). Acetate is predominantly produced by *Bifidobacterium* and *Bacteroides* spp.; propionate is primarily synthesized by *Bacteroides* and members of the Firmicutes phylum via the succinate pathway; while butyrate is chiefly derived from *Clostridium* spp. through the pyruvate fermentation pathway (88, 89). Beyond serving as a vital energy source for colonic epithelial cells, SCFAs act as potent immunoregulatory signaling molecules (69). Mechanistically, SCFAs contribute to asthma modulation through several pathways: By binding to G protein-coupled receptors GPR43 and GPR41, SCFAs activate downstream ERK1/2 and MAPK cascades, thereby influencing immune cell behavior and inflammatory signaling. They inhibit histone acetyltransferases and histone deacetylases, promoting epigenetic modifications that alter gene expression profiles relevant to inflammation and immune tolerance (90, 91). Furthermore, SCFAs help preserve airway mucosal integrity by enhancing epithelial barrier function, modulating T lymphocyte populations and activity, and downregulating proinflammatory cytokine production (92). Collectively, these actions contribute to the maintenance of gut–lung immune homeostasis, attenuation of airway inflammation, and suppression of asthma pathogenesis (**Supplementary Table 1**).

### 3.2 Lipopolysaccharides

Lipopolysaccharides (LPS), a potent pro-inflammatory molecule derived from the outer membrane of Gram-negative bacteria, are abundantly present within the human gut (93, 94). Functioning as a PAMP, LPS can bind to TLR4 expressed on dendritic cells, thereby activating downstream signaling cascades. This includes the stimulation of macrophages, activation of NF-κB, and subsequent release of large quantities of pro-inflammatory cytokines, chemokines, and ROS, collectively initiating and amplifying inflammatory responses that contribute to the pathogenesis of immune-mediated diseases such as asthma—characterized by chronic airway inflammation (95, 96). Notably, the immunological consequences of LPS exposure appear to be concentration-dependent. Low-dose LPS exposure preferentially induces a Th2-skewed immune response, characterized by elevated secretion of IL-5 and IL-13, which promotes eosinophilic inflammation. In contrast, high-dose LPS exposure favors Th17 polarization and the production of IL-17, leading to neutrophil recruitment and activation. The resulting increase in IL-17, IL-13, and IL-5 collectively drives both eosinophilic and neutrophilic airway inflammation, contributing to the development and exacerbation of asthma (97). However, paradoxically, early-life exposure to LPS has been shown to exert a protective effect. It reduces IL-13 levels, attenuates ROS production, and suppresses activation of the JAK2/STAT6 pathway. This inhibition leads to the upregulation of FOXA2, which downregulates MUC5AC expression and reduces mucus hypersecretion—thereby ameliorating asthma-related symptoms (98).

### 3.3 Tryptophan

Tryptophan, an essential amino acid required for normal growth and development, exists in two isomeric forms: L-tryptophan and D-tryptophan. While L-tryptophan is primarily derived from the diet, detectable levels of D-tryptophan in mammals are believed to originate from microbial metabolism within the gastrointestinal tract (99, 100). Tryptophan undergoes catabolism through three major pathways: the kynurenine pathway, the serotonin pathway, and the indole pathway. These metabolic routes give rise to a range of bioactive intermediates that regulate diverse physiological and pathological processes, including metabolism, immunity, neurotransmission, and inflammation (101, 102). A central enzyme in the kynurenine pathway is indoleamine 2,3-dioxygenase (IDO), which serves as a rate-limiting enzyme modulating immune tolerance (103, 104). Dendritic cells express IDO to inhibit T cell activation and promote the differentiation of naïve CD4^+^ T cells into CD4^+^CD25^+^ Tregs, thereby facilitating peripheral immune tolerance (105). In parallel, certain tryptophan metabolites exert immunoregulatory effects by inducing the expression of Foxp3, a master regulator of Treg development, while suppressing the expression of the retinoic acid receptor-related orphan receptor gamma (RORγ), a key transcription factor driving Th17 cell differentiation. This molecular skewing promotes Treg generation at the expense of Th17 lineage commitment (106). Moreover, several indole-derived tryptophan metabolites serve as natural ligands for the aryl hydrocarbon receptor (AhR). Upon activation, AhR signaling enhances epithelial barrier integrity and fortifies respiratory mucosal defenses against environmental insults and pathogens (107, 108). Additionally, D-tryptophan contributes to airway immune homeostasis by increasing gut microbial diversity and suppressing the growth of pathogenic bacteria, thus conferring protective effects against allergic airway inflammation (109).

## 4 Conclusion

Childhood asthma represents a multifactorial inflammatory disorder shaped by genetic predisposition, environmental exposures, and immune dysregulation. Growing evidence underscores the gut microbiota as a critical mediator linking early-life microbial colonization with long-term respiratory immune outcomes. Gut commensals influence key immunological processes, including Th1/Th2/Th17/Treg balance, dendritic cell maturation, and epithelial barrier integrity, thereby shaping host susceptibility to asthma. Moreover, microbial metabolites such as SCFAs, LPS, tryptophan derivatives, and secondary bile acids serve as potent immunoregulatory agents, capable of either promoting or attenuating airway inflammation depending on their context and concentration.

The gut–lung axis provides a conceptual and mechanistic framework through which intestinal microbial alterations influence pulmonary immunity. While significant progress has been made, several questions remain. In particular, the specific receptors, signaling nodes, and downstream transcriptional responses mediating microbiota–asthma interactions require further elucidation. Additionally, longitudinal and interventional studies in pediatric cohorts are urgently needed to determine whether modulation of the gut microbiota can serve as an effective therapeutic or preventive strategy. In conclusion, restoring gut microbial homeostasis and targeting key microbial metabolites represent promising avenues for alleviating asthma severity and improving pediatric respiratory health. Elucidating these microbe–immune crosstalk mechanisms hold significant translational potential for novel asthma management paradigms.
